# How the war on drugs impacts social determinants of health beyond the criminal legal system

**DOI:** 10.1080/07853890.2022.2100926

**Published:** 2022-07-19

**Authors:** Aliza Cohen, Sheila P. Vakharia, Julie Netherland, Kassandra Frederique

**Affiliations:** a Department of Research and Academic Engagement, Drug Policy Alliance, New York, NY, USA; b Drug Policy Alliance, New York, NY, USA

**Keywords:** Social determinants of health, war on drugs, criminalisation, surveillance, education, employment, substance use treatment, public benefits, child welfare, public policy, health policy

## Abstract

There is a growing recognition in the fields of public health and medicine that social determinants of health (SDOH) play a key role in driving health inequities and disparities among various groups, such that a focus upon individual-level medical interventions will have limited effects without the consideration of the macro-level factors that dictate how effectively individuals can manage their health. While the health impacts of mass incarceration have been explored, less attention has been paid to how the “war on drugs” in the United States exacerbates many of the factors that negatively impact health and wellbeing, disproportionately impacting low-income communities and people of colour who already experience structural challenges including discrimination, disinvestment, and racism. The U.S. war on drugs has subjected millions to criminalisation, incarceration, and lifelong criminal records, disrupting or altogether eliminating their access to adequate resources and supports to live healthy lives. This paper examines the ways that “drug war logic” has become embedded in key SDOH and systems, such as employment, education, housing, public benefits, family regulation (commonly referred to as the child welfare system), the drug treatment system, and the healthcare system. Rather than supporting the health and wellbeing of individuals, families, and communities, the U.S. drug war has exacerbated harm in these systems through practices such as drug testing, mandatory reporting, zero-tolerance policies, and coerced treatment. We argue that, because the drug war has become embedded in these systems, medical practitioners can play a significant role in promoting individual and community health by reducing the impact of criminalisation upon healthcare service provision and by becoming engaged in policy reform efforts.

**KEY MESSAGES**
- A *drug war logic* that prioritises and justifies drug prohibition, criminalisation, and punishment has fuelled the expansion of drug surveillance and control mechanisms in numerous facets of everyday life in the United States negatively impacting key social determinants of health, including housing, education, income, and employment.
- The U.S. drug war’s frontline enforcers are no longer police alone but now include physicians, nurses, teachers, neighbours, social workers, employers, landlords, and others.
- Physicians and healthcare providers can play a significant role in promoting individual and community health by reducing the impact of criminalisation upon healthcare service provision and engaging in policy reform.

## Introduction

Social determinants of health (SDOH) are “the conditions in the environments where people are born, live, learn, work, play, worship, and age that affect a wide range of health, functioning, and quality-of-life outcomes and risks.” [1] There is a growing recognition in the fields of public health and medicine that SDOH play a key role in driving health inequities and disparities, such that a focus on individual-level medical interventions will have limited effects without the consideration of the macro-level factors that dictate how effectively individuals can manage their health. For instance, differences in access to nutritious foods, safe neighbourhoods, stable housing, well-paying job opportunities, enriching school environments, insurance, and healthcare can lead to differential health outcomes for individuals, their families, and their communities. And as these mid- and downstream SDOH have gained more attention, we must also focus on more macro SDOH in order to understand “how upstream factors, such as governance and legislation, create structural challenges and impose downstream barriers that impact the ability and opportunity to lead a healthy lifestyle.” [2]

One underexplored upstream SDOH is the “war on drugs” in the United States and how it exacerbates many of the factors that negatively impact health and wellbeing, disproportionately affecting low-income communities and people of colour who already experience structural challenges including discrimination, disinvestment, and racism [3]. President Richard Nixon launched the contemporary drug war in the U.S. in 1971 when he signed the Controlled Substances Act and declared drug abuse as “public enemy number one.” [4] Since the declaration of the U.S. drug war, billions of dollars each year have been spent on drug enforcement and punishment because it was made a local, state, and federal priority [5]. For the past half century, the war on drugs has subjected millions to criminalisation, incarceration, and lifelong criminal records, disrupting or altogether eliminating access to adequate resources and supports to live healthy lives.

Drug offences remain the leading cause of arrest in the nation; over 1.1 million drug-related arrests were made in 2020, and the majority were for personal possession alone [6]. Black people – who are 13% of the U.S. population – made up 24% of all drug arrests in 2020, despite the fact that people of all races use and sell drugs at similar rates [6–8]. While incarceration rates for drug-related offences skyrocketed in the 1980s and 1990s, they have decreased in recent years motivated both by cost savings and criminal legal reform efforts to promote a public health approach to drug use. However, estimates still suggest that roughly 20% of people who are incarcerated are there for a drug charge, and racial disparities in incarceration persist [9,10].

Meanwhile, the illicit drug supply has become increasingly unpredictable and contaminated due to drug supply disruptions, contributing to an exponential increase in drug overdose deaths [11,12]. Estimates suggest that one million people died of a drug-involved overdose between 1999 and 2020, with over 100,000 deaths occurring in a calendar year for the first time in 2021 [13,14]. Since 2015, overdose deaths have disproportionately impacted racial and ethnic minorities; Black people have had the biggest increase in overdose fatality rates, and today, Black and Native people have the highest overdose death rates across the U.S [15]. The most recent “fourth wave” of the overdose crisis can be attributed to a fentanyl-contaminated drug supply caused by drug prohibition; criminalisation that leads to stigma and fear of punishment that deters people from getting support they might need; and a lack of robust, scaled-up investment in harm reduction and evidence-based treatment services [16,17]. Although harm reduction interventions, including supervised consumption spaces (also called supervised injection facilities, drug consumption rooms, or overdose prevention centres) and heroin-assisted treatment have been widely studied and found effective outside of the U.S., these strategies have not been widely adopted in this country [18–21].

The drug war has also become deeply embedded within many of the systems and structures of U.S. life well beyond the criminal legal apparatus [3]. Since the health impacts of incarceration have been studied elsewhere, this paper will specifically discuss the impacts of criminalisation in other facets of life [22].

We argue that an underlying *drug war logic* has fuelled the expansion of drug surveillance and control mechanisms in numerous facets of everyday life in the U.S. We define *drug war logic* as a logic that prioritises and justifies drug prohibition, criminalisation, and punishment to purportedly address the real and perceived health harms of drug use over a public health approach to address these issues. In coining this term, we hope to make more visible the implicit assumptions about drug use that are often unnamed but common in the policies and practices across different institutions. We acknowledge that many actors in these settings where drug war logic is embedded, including physicians and other healthcare providers, are often well-intentioned yet unaware of how they may be perpetuating this logic through their own actions. We argue that drug war logic defies and contradicts widely accepted understandings of addiction as a health issue and has, in many cases, made a public health approach more challenging to implement [23]. Notably, the American Society of Addiction Medicine defines addiction as “a treatable, chronic medical disease involving complex interactions among brain circuits, genetics, the environment, and an individual’s life experiences.” [24] As this paper will outline, drug war logic undermines rather than supports the health of people who use drugs, their families, and their communities by treating drug use as a criminal issue.

Drug war logic is made concrete, not just within criminal legal systems, but also through mandated drug reporting and monitoring systems in treatment and healthcare settings, compulsory drug testing in employment and for the receipt of social services, the proliferation of zero-tolerance workplaces and school zones, mandated treatment in order to receive resources or avoid loss of benefits, background checks for work and housing, and numerous other measures which will be discussed in detail below. As a result, the drug war’s frontline enforcers are no longer police alone but now include physicians, nurses, teachers, neighbours, social workers, employers, landlords, and others who are required to engage in these forms of surveillance and punishment.

This commentary will use a SDOH lens to explore a number of systems where the drug war and its logic have taken root, impacting individual and community health and subjecting many people in the U.S. to surveillance due to suspected or confirmed drug use. Healthcare providers must have a robust understanding of the impact of drug war logic in employment, housing, education, public benefits, the family regulation system (commonly referred to as the child welfare system), the drug treatment system, and the healthcare system because these deeply impact the health of their patients, particularly their patients who use drugs (For the purposes of this paper, we are using the term “Family Regulation System,” coined by Emma Williams and used by other scholars, instead of the more commonly used term “Child Welfare System” to reflect the fact that, particularly for low-income families and families of color, state intervention often occurs in order to regulate their families rather than to prioritize the welfare of the entire family unit, of which the child is a part).

## Employment

Employment, with its link to income and health insurance, is an important determinant of health. However, drug testing, criminal background checks, and exclusions of those with criminal histories from certain professions create significant barriers to obtaining and maintaining employment. Beginning in the 1980s, employment-based drug testing became widespread. In a 1994 report, the National Research Council noted that “[i]n a period of about 20 years, urine testing has moved from identifying a few individuals with major criminal or health problems to generalized programs that touch the lives of millions of citizens.” [25] Between 2017 and 2020, the National Survey on Drug Use and Health found that approximately 21% of respondents were tested as part of the hiring process, and 15% were subject to random employee drug testing [26].

Despite the widespread use of testing, less than 5.5% of results are positive for any drug, according to data from Quest Diagnostics, one of the largest testing companies in the country [27]. There is little evidence that these policies are effective in reducing drug use, improving workplace safety, or increasing productivity [28–30]. Notably, drug tests cannot specify how much of a drug was consumed, whether the person is currently intoxicated or impaired, or if they have a SUD. Drug tests cannot indicate if drug use will impact a person’s ability to perform their work or if they present a safety risk. Rather, drug tests simply show whether or not someone has a particular metabolite in their system [31–35].

Beyond workplace drug testing, hundreds of thousands are excluded from stable, well-paid work because of drug-related convictions. Over 70 million people – more than 20% of the U.S. population – have some type of criminal record [36]. A drug arrest or charge, even without a conviction, can be a barrier to getting a job because it can appear in many web searches and background checks [37]. Criminal background checks have become cheaper and easier to access, even though these records are notoriously inaccurate [38,39]. In addition, more than a quarter of jobs in the U.S. require some kind of licence, and a drug conviction history can automatically prevent people from getting a professional licence for their trade, like trucking or barbering [40].

These employment barriers disproportionately affect Black men, who already face additional impediments to employment and who are most harmed by the drug war and criminalisation [41]. The federal Equal Employment Opportunity Commission issued guidance stating that denying employment based on criminal records could be a form of racial discrimination because people of colour are more likely to be targeted by law enforcement and thus more likely to have an arrest or conviction record [42,43]. As a recent report by the Brennan Centre points out: “the staggering racial disparities in our criminal justice system flow directly into economic inequality” [36]. This same report found that those with a history of imprisonment earned 52% less than those with no history of incarceration.

Employment is a health issue that should be of concern to healthcare providers because it provides income, access to health insurance and medical treatment, and social connection [44]. Precarious employment and low income are linked to poor health, and some research has shown that people who use drugs and who are precariously employed face increased vulnerability to violence and HIV infection [45–47]. Being unemployed can lead to poverty and negative health effects and is associated with increased rates of drug use and SUDs [48].

Rather than supporting people who use drugs in accessing employment and the health benefits attached to it, drug war logic in employment settings can erect barriers. Eliminating or greatly restricting workplace drug testing as well as banning criminal background checks and professional licencing restrictions are important steps towards restoring access to employment and the many health benefits it confers.

## Housing

Housing is another key SDOH that is significantly impacted by drug war policies and practices. Drug war surveillance in housing began with the passage of the Anti-Drug Abuse Act of 1988, which prohibited public housing authorities (PHAs) from allowing tenants to engage in drug-related activity on or near public housing premises and deemed such activity grounds for immediate eviction [49].

The Cranston-Gonzalez National Affordable Housing Act of 1990 expanded on this so that if a tenant’s family member or guest - regardless of whether they live on-site - engages in drug-related activity, the tenant and their household can be evicted [50]. Additionally, the Act states that evicted households must be banned from public housing for a minimum of three years unless the tenant completes an agency-approved drug treatment program or has otherwise been “rehabilitated successfully.” [50]

Six years later in 1996, Congress passed the Housing Opportunity Program Extension Act, which established “One Strike” laws and expanded on previous acts to give PHAs the authority to evict tenants if they or a guest was suspected of using or selling drugs, even *outside* of the premises [51]. This series of public housing policies requires neither a drug arrest nor proof that a tenant or their guest is involved in drug use, sales, or activity [52].

Private housing markets can also enforce zero-tolerance drug policies. In over 2,000 cities across the U.S., landlords can certify their property as “crime-free” by taking a class, implementing “crime prevention” architecture, and including clauses in their leases that allow for immediate eviction should a tenant, family member, or guest engage in “criminal activity,” particularly drug-related activity, on or off the premises [53,54]. Landlords, in close partnership with law enforcement, can invoke these laws by claiming to enforce crime-free ordinances, regardless of whether the alleged drug-related activity is illegal. In states across the U.S., private landlords have evicted tenants following an overdose [55–59]. In practice, these programs and ordinances increase the surveillance and displacement of low-income Black and Latinx tenants while not decreasing crime and potentially deterring someone from calling 911 for medical assistance in case of an overdose [55].

Evictions can lead to unstable housing or homelessness, which is associated with a host of chronic health problems, infectious diseases, emotional and developmental problems, food insecurity, and premature death [60–63]. Lacking a permanent address and reliable transportation makes it more difficult to receive and store medications and travel to a hospital or clinic; this is compounded with the stigma and discrimination that unhoused people often face from healthcare providers [64]. Being unhoused or housing unstable is also associated with difficulty obtaining long-term employment and education [65–67]. Longitudinal studies have found that family eviction has both short- and long-term impacts among newborns and children, including adverse birth outcomes, poorer health, risk of lead exposure, worse cognitive function, and lower educational outcomes [68]. These negative health outcomes are compounded for people with SUDs [69]. Unhoused people who use drugs are often forced into more unsafe, more unsanitary, and riskier injection and drug-using practices to avoid detection [70]. Evictions and homelessness are also associated with increased risk of drug-related harms, including non-fatal and fatal overdose, infectious diseases, and syringe sharing [71–73]. In addition, evictions can disrupt relationships between users and trusted sellers, making an already unregulated drug supply even more unpredictable [70].

While housing is understood as a key component of health and safety for all people, including people who use drugs, drug war logic can encourage and facilitate displacement, making it hard for housed people to remain so and creating barriers for those who are unhoused to find safe, affordable housing options. Solutions for improving housing access include ending evictions and removing housing bans based solely on drug-related activity or suspected activity, restricting landlords from using criminal background checks to exclude prospective tenants, and ending collaborations between housing complexes and law enforcement. Housing interventions that can improve the health of people who use drugs, in particular, include investing in Housing First programs and permanent supportive housing, providing eviction protection to people who call for help during an overdose emergency (i.e. expanding 911 Good Samaritan laws), and establishing overdose prevention centres.

## Education

Education is also understood as a strong predictor of health [74–76], but drug war logic in educational settings can subject young people who use drugs to punishment rather than needed support. Adolescent substance use is associated with sexual risk behaviour, experience of violence, adverse childhood experiences, and mental health and suicide risks, which should justify greater mental health and support services in schools [77]. Despite this, punitive responses to suspected or confirmed drug use, ranging from surveillance and policing to drug testing and expulsion, are commonplace in the field of education.

In 2018, 94% of high schools used security cameras, 65% did random sweeps for contraband, and 13% used metal detectors [78]. Twenty-four states and the District of Columbia have almost as many police and security officers in schools as they do school counsellors [79,80]. Drug use is one of the most common sources of referrals of students to police [80]. And recent estimates show that over a third of all U.S. school districts with middle or high schools had student drug testing policies [81–83].

Drug war policies also impact higher education, which is integral to economic mobility [84]. Prior to December 2020, federal law prohibited educational grants and financial aid to people in prison, one-fifth of whom were there for a drug offence, and drug convictions could lead to temporary or indefinite suspension of federal financial aid for students [85]. Still today, fourteen states have some temporary or permanent denial of financial aid for college or university education for people with criminal records [86].

These education policies – surveillance, policing, drug testing, zero tolerance, and barriers to financial aid – restrict access to education and ultimately impede economic wellbeing and positive health outcomes. For example, dropout risk increases every time a student receives harsh school discipline or comes into contact with the criminal legal system, including through school police officers [87]. Dropping out, in turn, is associated with higher unemployment and chronic health conditions [88]. In addition, discipline, such as expulsion for a drug violation, can contribute to more arrests for drug offences or the development of SUDs [89–91]. In contrast, school completion can help reduce higher risk substance use patterns [92], and education is a strong predictor of long-term health and quality of life [93].

Rather than supporting young people in completing their education and getting the support they may need, drug war logic prioritises punishing them in schools while often restricting access to financial aid and educational services for those seeking higher education. If we want to improve the health of young people, we need to reverse these policies. For example, the American Academy of Paediatrics opposes the random drug testing of young people based on an exhaustive review of the literature finding it did more harm than good [94]. Removing police from schools, ending zero-tolerance policies, and offering young people who use drugs counselling and support, instead of expulsion, could also help improve completion rates, ultimately leading to better health outcomes.

## Public benefits

Though economic and food insecurity are linked with poor health outcomes, decades of drug policies have restricted access to public assistance programs. In 1996, Congress passed the Personal Responsibility and Work Opportunity Reconciliation Act (PRWORA) [95], and one of the stated goals was to facilitate the transition from reliance on public assistance to full-time employment [96]. This law restricted benefits for people who use drugs, people with prior drug convictions, and their families in several ways.

The PRWORA introduced a lifetime ban on Supplemental Nutrition Assistance Program (SNAP) and Temporary Assistance for Needy Families (TANF) cash assistance benefits for people with felony drug convictions, unless the state modified or opted out of the ban. Today, one state - South Carolina - fully bars people with felony drug convictions from receiving SNAP, and twenty-one states have instituted a modified SNAP ban [97]. Seven states fully bar people with felony drug convictions from receiving TANF, and seventeen states and the District of Columbia have instituted modified TANF bans [97]. Common features of modified bans can include mandatory drug treatment, drug testing, and parole compliance [98,99]. These zero-tolerance bans have discriminatory and disproportionate impacts among Black and Latinx people and women, who are disproportionately incarcerated for federal and state drug offences [100].

Drug testing of public benefits applicants is less discussed in the peer-reviewed literature [101]. Although the PRWORA authorised, but did not require, drug screenings of public benefits applicants, today 13 states drug test TANF applicants [102,103]. States that drug test as a condition of receiving TANF can only test if drug use is suspected. For example, some states automatically require people with felony drug convictions to take a drug test [104], while other states require all applicants to undergo a drug screening questionnaire and then require a test if there is suspicion of drug use [105]. Many TANF applicants, who are already low income, are expected to pay for their drug tests. The impact of drug testing on people with felony drug convictions is compounded since they are already disproportionately poor, unemployed, and food insecure compared to people who have never been incarcerated [106–108].

In most states that test, a positive drug test can temporarily or permanently disqualify a person from receiving TANF benefits [105]. Even if cash assistance is allocated to other household members (e.g. children) through a different parent or guardian, overall benefits for the family can be reduced. In some cases, a person who tests positive for drugs may still receive benefits but only if they complete mandated, abstinence-based treatment [105]. Such policies and practices can deter many eligible candidates and those in need of support from ultimately seeking these public benefits altogether [109].

There are numerous negative health consequences associated with food and economic insecurity [110–112]. In particular, studies have found that loss or reduction of SNAP is associated with increased odds of household and child food insecurity and increased odds of forgoing health or dental care [113]. Loss or reduction of TANF is associated with increased risk of hunger, homelessness or eviction, utility shutoff, inadequate medical care, and poor health [114].

When people are seeking financial and nutritional support to better care for themselves and their families, especially in crisis, drug war logic justifies more barriers to SNAP and TANF and the discontinuation of assistance precisely when people need it the most. To better support financial and economic security of low-income people, advocates can support removing TANF and SNAP bans for people who have felony drug convictions, ending drug testing requirements for public assistance, eliminating mandatory drug treatment requirements for public benefits applicants and recipients, and adequately investing in public benefit programs to ensure they provide enough assistance for families.

## Family regulation

The family regulation system (FRS) often treats any drug use as a predictor of child abuse or neglect, even though research shows that poverty is one of the largest predictors of adverse infant and child health outcomes [115]. Drug war logic within the FRS justifies the separation and punishment of families for drug use even absent evidence of abuse or neglect. Half of all states and the District of Columbia require healthcare professionals to report any suspected drug use during pregnancy to FRS authorities, and eight states require them to drug test patients suspected of drug use [116]. Statutes in nineteen states and the District of Columbia define any drug use during pregnancy as a form of child maltreatment [117]. These policies exist even though most people who use drugs use them infrequently and do not meet criteria for SUDs [118]. Additionally, evidence proving causal links between prenatal drug use and child harm and maltreatment is limited. Research finds that in utero exposure to drugs may not have long-term negative developmental impacts on the child and that confounding variables, like poverty and food insecurity, have significant and often stronger impacts on child development than drug use [117].

Drug testing, mandatory reporting, and the prospect of punishments result in poorer health outcomes for pregnant people who use drugs, especially if they struggle with their use. A fear of punishment and family separation leads some pregnant people who use drugs to avoid honest, open conversations about healthcare needs or how to reduce drug use harms so that many delay, avoid, or forgo prenatal care altogether [119,120].

Like healthcare professionals, most school teachers, counsellors, social workers, and mental healthcare providers are required by law to report any suspicion of child maltreatment or neglect, which then initiates an FRS investigation [121]. A child can be removed from their home if the caregiver tests positive for drugs, even absent any other evidence of mistreatment or abuse. In addition, a positive drug test can lead to a parent being mandated to complete abstinence-based treatment even if the parent does not meet criteria for a diagnosable SUD [122]. Intervention by the FRS, such as placing children in foster care, can lead to adverse education, employment, and mental and behavioural health outcomes among children; increased parental mental illness diagnoses; and increased parental drug use to cope with the trauma of family separation [123–125].

These policies have disproportionate impacts on Black people. Black pregnant women are more likely to be tested for drug use, and Black women are reported to the FRS at higher rates than white women [126–128]. Over half of Black children will experience an FRS investigation at some point during their lifetime [129]. One study that analysed cumulative foster system removals between 2000 and 2011 found that 1 in 17 U.S. children, 1 in 9 Black children, and 1 in 7 Indigenous children will experience foster placement before they turn 18, and data show that many FRS cases involve allegations of parental drug use at some point [130]. These disparities in FRS involvement are not because Black parents are using drugs or mistreating their children at higher rates; rather, it’s because Black families, especially poor Black families, more often encounter state systems – like public hospitals and public benefits offices – and mandated reporters within these systems that monitor behaviour and drug use [131].

Drug war logic prioritises separation, coercion, and punishment in families where drug use occurs or is suspected. For pregnant people and parents who do use problematically, their use should be treated as a public health issue, according to international bodies like the United Nations General Assembly Special Session on drugs [132]. Advocates can support legislative policy changes to prohibit removals based on drug tests alone, eliminate mandatory reporting for drug use alone, and repeal laws that define drug use during pregnancy as de facto child abuse or maltreatment. Healthcare professionals can also advocate to only allow drug testing when medically necessary and when the parent provides informed consent; support practices that keep parents and infants together, like breastfeeding and skin-to-skin contact, that can mitigate the effects of neonatal abstinence syndrome [133,134]; and create programs providing both perinatal healthcare and SUD treatment to improve access and continuity of care as well as initiation and maintenance of medications for addiction treatment.

## Substance use treatment system

Substance use treatment can be an essential lifeline for people with SUD working towards recovery. Yet surveillance and punishment are embedded into SUD treatment through the numerous constraints placed upon clients because of the role of institutional referral sources in treatment, such as the criminal legal system, the FRS, social services, and others. Studies suggest that roughly 25% of clients in publicly funded treatment were referred from the criminal legal system as a condition of their probation, parole, or drug court program [135]. This has led to therapeutic jurisprudence: the belief that the criminal legal system can support and facilitate efforts towards rehabilitation using the threat of incarceration [136]. Another 25% of clients are referred to treatment by other sources, including the FRS, social services, schools, and employers [133]. Criminal legal controls such as those from the courts, or formal social controls such as those from the other aforementioned institutions, coerce clients to either comply with treatment or face other harsh consequences, like incarceration, the termination of parental rights, or losing public benefits [137].

Treatment providers monitor client compliance and abstinence by conducting and observing routine urine drug tests, and providers are often in regular contact with referral sources about client progress in treatment. Any drug use or negative progress reports can be used as grounds to sanction those on probation, parole, or in drug court which can lead to incarceration and, in cases of drug courts, longer sentences than if participants had accepted a jail sentence [136]. Clients referred by other sources can also face ramifications for positive drug tests or treatment non-compliance, impacting child custody hearings as well as their ability to secure certain social services and resources, stay enrolled in school, or remain employed.

Referral sources influence the type of care that clients receive in facilities, including evidence-based treatments. Research suggests that only 5% of clients with opioid use disorder (OUD), who were referred to treatment from the criminal legal system, received either methadone or buprenorphine, compared to nearly 40% those who were not referred by the system [138]. This represents an extension of a broader problem within the criminal legal system wherein access to these gold standard medications for OUD is almost nonexistent in most jails and prisons across the U.S [139].

Drug war logic is also deeply rooted in the restrictions for prescribing and dispensing methadone and buprenorphine since they are controlled substances under the oversight of the Drug Enforcement Agency, a federal law enforcement entity. When taken in effective doses, these life-saving medications can cut the risk of overdose and all-cause mortality dramatically among people with OUD [140]. However, due to tight federal restrictions and guidelines for these controlled medications, patients can be subjected to routine drug testing, counselling requirements, daily clinic visits, and observed or highly monitored medication dispensing. Patients deemed non adherent to medications or who test positive for other drugs can then be subjected to dose reductions, required to attend treatment more frequently, or even terminated from care altogether [141]. The tight restrictions on both methadone and buprenorphine, combined with the oversight of the DEA, create obstacles for prescribers and stigmatise these medications by conveying that they cannot be used like other medications in routine healthcare [142]. These policies have also contributed to striking racial disparities in who receives buprenorphine versus methadone due to costly co-pays and insurance coverage issues [143]. Studies also suggest that the DEA’s involvement in monitoring buprenorphine has made pharmacies reluctant to stock the medication or to dispense it to patients for fear of triggering an investigation [144,145]. Ultimately, it is estimated that only 10% of all people with OUD receive these medications [146].

Providers can take steps to extract the drug war from our substance use treatment system, through their conscious and judicious documentation of treatment progress since those records could be used by criminal legal and other referral sources in decisions about clients and their families. In addition, eligible buprenorphine prescribers should begin prescribing to patients and join advocacy efforts to change policies to expand access to buprenorphine and methadone through looser restrictions.

## Healthcare system

People with SUDs often have high rates of co-occurring medical needs requiring treatment, including psychiatric disorders, infectious diseases, and other chronic health conditions. However, research suggests that people with SUDs are often deterred from seeking healthcare to address their medical needs due to prior negative and stigmatising experiences with providers, and that having experienced discrimination in healthcare is associated with greater risk behaviours, psychological distress, and negative health outcomes among people who use drugs [147–149]. Some of these challenges are due to a lack of training on how to work with patients with SUDs, in addition to pre-existing personal biases and stigmatising views held by healthcare professionals, which impacts the type of care they provide [142].

The widespread use of drug testing in healthcare settings also creates ethical challenges and conflicts for providers and patients since results are often entered into the electronic health record (EHR). While EHRs are typically thought of as beneficial and intended for greater transparency and access, they also pose challenges surrounding patient privacy, confidentiality, and autonomy; they can, therefore, make patients reluctant to disclose drug use or consent to drug testing [150]. For instance, medical records that include drug test results, can be accessed by a wide variety of actors in the medical system, subpoenaed for court, and used in future medical decision making without the patient’s knowledge or consent. Providers might not receive adequate training to weigh the need for these tests as part of treatment adherence monitoring with the potential social or legal ramifications of these tests for the patient. Patients might also not be adequately informed of these potential consequences prior to testing.

Universal drug screening and testing in obstetric and gynecological care is an example wherein testing intersects with the role of most healthcare providers as mandated reporters. Mandated reporting for suspected child abuse or neglect due to parental drug use is purported to protect the foetus or children in the parents’ custody, yet this can often be a deterrent for patients to seek medical treatment altogether if they believe that they may lose their children or be subject to other mandates. The racial and class disparities in how such testing is used, as well as the punitive measures used against families, have been noted earlier in the text but is a compelling reason for healthcare providers to consider making recommendations for counselling or supportive case management in order to address family challenges.

Healthcare providers need more training and resources to work with patients with SUDs to ensure that they are engaging them in evidence-based treatments and treating their complex medical needs while avoiding some of the lifelong and harmful ramifications that can occur when drug testing, health records, and mandated reporting deter patients from seeking and receiving care.

## Conclusion

Because of the social, economic, and health effects of drug policies, the work of ending the drug war cannot be situated within criminal legal reform efforts alone. The drug war and a punitive drug war logic impact most systems of everyday life in the U.S., subjecting people to surveillance, suspicion, and punishment and undermining key SDOH, including education, employment, housing, and access to benefits. Combined, these have resulted in poorer health outcomes for individuals, families, and communities, particularly for people who use drugs. These policies and practices, while race-neutral as written, are not [151]. The targeted effects on people of colour further entrench health and economic disparities. As the public and policymakers call for a health approach to drug use, it is vital to recognise how systems meant to care and support are often unable to serve their intended purposes; rather than help people who use drugs or are suspected of using drugs, they frequently punish them.

In their day-to-day practice, healthcare professionals must understand the deep roots of the drug war as well as their role in both perpetuating and undermining drug war logic and practices. Healthcare providers can treat people who use drugs with dignity, respect, and trust and ensure that healthcare and treatment decisions are made in partnership with individuals. Medical professionals can also work to situate drug use within a larger social and economic context [152], understanding that drug-related harms often stem from lack of resources – like housing and food precarity, economic insecurity, and insufficient healthcare – rather than from drugs themselves. Treatment need not be the only antidote for people who experience drug-related harms but should be one option among an array of health services, resources, and support.

At the mezzo- and institutional levels, healthcare providers can advocate to shift hospital and programmatic policies around drug testing, mandatory reporting, and collaborations with law enforcement. As outlined in this paper, drug testing is not an effective monitoring strategy for care and support, but rather, it is more often a punitive tool of surveillance. If drug testing cannot be eliminated, at the very least, patients should have the right to understand the implications of drug testing and provide explicit consent for the test. To the extent possible, providers should not share private patient information with police or state agencies. Healthcare professionals should understand the implications of reporting positive drug tests and suspicion of use and should work to change these policies where possible and inform their patients of them. Providers can ensure that their patients who use drugs have access to evidence-based, non-coercive harm reduction and treatment options in addition to robust and supportive primary healthcare. Healthcare professionals involved with medical education and licensure can work to ensure that all students graduate with a deep understanding of SDOH and the impact of the drug war on individual and community health.

Finally, healthcare providers can get involved with policy-level changes to end drug testing, mandatory reporting, zero-tolerance policies, coerced treatment, and denial of services and resources based on arrest or conviction records at the municipal, state, and federal levels. Providers can follow the leadership and expertise of people who use drugs, some of whom have organised themselves into user unions [153]. Policy advocacy can include drafting and joining sign-on letters, delivering expert testimony, speaking to media, writing op-eds, and lobbying medical professional organisations to release policy statements. Providers, who see firsthand the consequences of the war on drugs, are well positioned to be effective advocates in undoing these harmful policies that have for too long undermined key SDOH [154]. In order to improve individual and collective health, healthcare providers should resist drug war logic and work to transform these systems so they can truly promote health and safety.

For the purposes of this paper, we are using the term “Family Regulation System,” coined by Emma Williams and used by other scholars, instead of the more commonly used term “Child Welfare System” to reflect the fact that, particularly for low-income families and families of color, state intervention often occurs in order to regulate their families rather than to prioritize the welfare of the entire family unit, of which the child is a part.

## Authors contribution

All authors (AC, SV, JN, KF) were involved in the conception and drafting of the paper, revising it critically for intellectual content; and the final approval of the version to be published. All authors agree to be accountable for all aspects of the work.

## Data Availability

Data sharing is not applicable to this article as no new data were created or analysed in this study.
